# A high-resolution integrated map of copy number polymorphisms within and between breeds of the modern domesticated dog

**DOI:** 10.1186/1471-2164-12-414

**Published:** 2011-08-16

**Authors:** Thomas J Nicholas, Carl Baker, Evan E Eichler, Joshua M Akey

**Affiliations:** 1 Department of Genome Sciences, University of Washington, 1705 NE Pacific, Seattle, WA. 98195, USA; 2 Howard Hughes Medical Institute, Seattle, WA, USA

## Abstract

**Background:**

Structural variation contributes to the rich genetic and phenotypic diversity of the modern domestic dog, *Canis lupus familiaris*, although compared to other organisms, catalogs of canine copy number variants (CNVs) are poorly defined. To this end, we developed a customized high-density tiling array across the canine genome and used it to discover CNVs in nine genetically diverse dogs and a gray wolf.

**Results:**

In total, we identified 403 CNVs that overlap 401 genes, which are enriched for defense/immunity, oxidoreductase, protease, receptor, signaling molecule and transporter genes. Furthermore, we performed detailed comparisons between CNVs located within versus outside of segmental duplications (SDs) and find that CNVs in SDs are enriched for gene content and complexity. Finally, we compiled all known dog CNV regions and genotyped them with a custom aCGH chip in 61 dogs from 12 diverse breeds. These data allowed us to perform the first population genetics analysis of canine structural variation and identify CNVs that potentially contribute to breed specific traits.

**Conclusions:**

Our comprehensive analysis of canine CNVs will be an important resource in genetically dissecting canine phenotypic and behavioral variation.

## Background

The domestication of the modern dog from their wolf ancestors has resulted in an extraordinary amount of diversity in canine form and function. As such, dogs are poised to provide unique insights into the genetic architecture of phenotypic variation and the mechanistic basis of strong artificial selection. A number of canine genomics resources have been developed to facilitate genotype-phenotype inferences, including a high-quality whole genome sequence and a dense catalog of SNPs discovered in a wide variety of breeds [1-3]. These genomics resources have been successfully used to identify an increasing number of genes that influence hallmark breed characteristics such as size, coat texture, and skin wrinkling [4-6]. Additionally, SNP data has been used to investigate patterns of genetic variation within and between breeds, establish timing and geography of domestication, examine relatedness among breeds, and identify signatures of artificial selection [4,7-9].

In addition to SNPs, it is important to characterize additional components of canine genomic variation in order to comprehensively assess the genetic basis of phenotypic diversity. For example, structural variation in general, and copy number variants (CNVs) in particular, has emerged as an important source of genetic variation in a wide range of organisms including dogs [10-18]. Duplications and deletions of genomic sequence can have significant impacts on a wide range of phenotypes including breed-defining traits. For example, a duplication of a set of *FGF *genes in Rhodesian and Thai Ridgebacks leads to the breeds characteristic dorsal hair ridge [19].

Although the *FGF *duplication provides a vivid example of the phenotypic consequences of structural variation in dogs, it remains unknown whether CNVs are an appreciable source of variation in morphological, behavioral, and physiological traits within and between breeds. Comprehensive discovery of structural variation in a diverse panel of breeds is an important first step in more systemically delimiting the contribution of CNVs to canine phenotypic variation. Previously, we used a customized aCGH chip to identify nearly 700 CNV regions located in segmental duplications (SDs) [17]. However, SDs only cover approximately 5% of the dog genome and thus a large fraction of total genomic space was unexplored. An additional study using a genome-wide tiling array from NimbleGen identified approximately 60 CNV regions outside of SDs [10]. However, the low probe density (~1 probe every 5 kb), limited the number and size of CNVs that could be identified.

In an effort to more comprehensively interrogate the canine genome for CNVs, we used a high-density (~1 probe every 1 kb) genome-wide tiling array to discover additional CNVs in a panel of nine genetically and phenotypically diverse dogs. In total, we discover over 400 new CNV regions. Moreover, we designed a custom aCGH chip to genotype all known canine CNVs in 61 dogs from 12 diverse breeds, allowing the first population genetics analysis of structural variation in dogs to be performed. The comprehensive CNV resources that we have developed will be important tools in genetically dissecting canine phenotypic variation.

## Results and Discussion

### Genome-wide identification of CNVs using a high-density aCGH chip

We performed aCGH using a high-density tiling array in nine breeds (Table 1), a gray wolf, and a self-self hybridization. These nine breeds and gray wolf samples were previously studied using a custom array that exclusively targeted regions containing SDs [17]. In all of the aCGH hybridizations we used the same reference sample (a female Boxer distinct from Tasha, the Boxer used for generating the canine reference sequence), which was also the reference in our prior SD experiments [17]. The aCGH chip consists of over 2.1 million probes distributed across the genome (not including the uncharacterized chromosome, chrUn) with an average probe density of 1 kb. CNVs were identified using a circular binary segmentation algorithm implemented in the program segMNT, part of NimbleGen's NimbleScan software package. These calls were filtered by log_2 _values and number of probes using an adaptive threshold algorithm where the specific filtering criteria were a function of the size of the CNV (see Methods).

**Table 1:** Summary of CNVs identified with the genome-wide aCGH chip

	Number of CNVs				
		
Breed	Total	Gain	Loss	Average Size (kb)	Genes
Basenji	109	45	64	54.9	114
Doberman Pinscher	107	57	50	83.8	88
German Shepherd	113	52	61	88.2	105
Labrador Retriever	77	33	44	90.9	88
Pug	97	44	53	62.3	74
Rottweiler	88	30	58	92.6	65
Shetland Sheepdog	86	35	51	123.5	91
Siberian Husky	86	47	39	61.7	91
Standard Poodle	109	37	72	64.6	127
Wolf	136	79	57	86.5	127
Self	0	0	0	0	0

**Average**	**101**	**46**	**55**	**80.9**	**97**

We identified 1,008 CNVs in 403 unique CNV regions spanning 30.5 Mb of genomic sequence (Table 1). In the self-self hybridization, no CNVs were called using the same analysis and filters. The average number of CNVs per individual was 101, ranging from 86 (Shetland Sheepdog and Siberian Husky) to 136 (Gray Wolf). The average CNV size was approximately 81 kb (Table 1), and the largest CNV region was located on CFA 34 and spans 3.9 Mb. In total, these 403 CNV regions overlap or contain 401 protein coding genes. After assigning all genes PANTHER Molecular Function terms, we found that the most enriched gene classes are similar to those identified in SDs, namely, defense/immunity, and receptor genes, but also included oxidoreductase, protease, signaling molecule, and transporter genes (Additional file 1).

**Figure 1:**
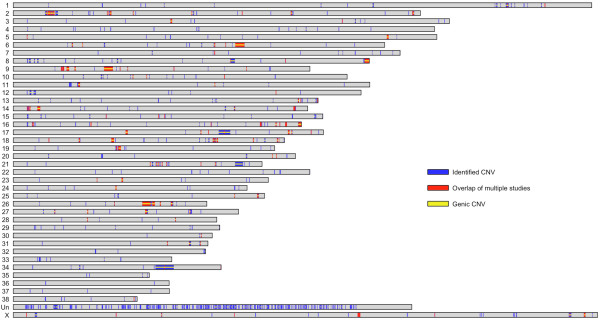
**An integrated map of all known CNVs in the canine genome**. Gray bars represent chromosomes. Blue marks indicate the locations of 910 identified CNV regions. Red marks CNV regions that have been found in at least two different studies. Yellow stripes in the middle of the chromosomes mark CNV regions that contain or overlap known and predicted genes.

Figure 1 summarizes the location and characteristics of all known dog CNVs derived from this and previous studies [10,17]. In total, after merging closely spaced CNVs, 910 distinct CNV regions that cover over 49.8 Mb have been identified. Of these regions, 395 contain or overlap protein coding genes and 134 have been found in multiple experiments. Larger CNVs were more likely to be observed in multiple studies (average size of CNVs identified in multiple versus single studies was 220 kb versus 64 kb, respectively). As expected, the uncharacterized chromosome (ChrUn), consisting of sequences that cannot be uniquely mapped to the genome, is particularly enriched for CNVs as it harbors approximately 65% of segmental duplications [17], which are hotspots of CNV formation.

### Comparison of SD vs Non-SD CNVs

We used the same individuals and reference sample as in our previous study of CNVs in segmental duplications, providing an opportunity to directly compare characteristics of CNVs between SDs and non-SD regions (Table 2). While most CNVs were not associated with SDs, on average CNVs associated with SDs were much larger (160.1 kb vs 33.6 kb; Table 2) resulting in the majority of CNV space to be associated with SDs (21.5 Mb or 70%). Similarly, the majority of genic CNVs were also found in CNVs associated with SDs (66%).

**Table 2:** Comparison of CNVs located in SDs and outside of SDs

Breed	CNV Location	Gain	Loss	Complex	Singletons	Average Size (kb)	Genes
Basenji	SD	17	22	3	4	173.0	82
	non-SD	17	38	0	23	35.5	32
Doberman Pinscher	SD	21	16	5	3	144.2	59
	non-SD	22	25	0	15	40.8	29
German Shepherd	SD	21	28	1	6	220.5	87
	non-SD	26	29	0	22	40.2	18
Labrador Retriever	SD	15	18	1	2	184.2	48
	non-SD	14	23	0	14	91.8	40
Pug	SD	11	18	4	7	223.1	42
	non-SD	25	25	0	20	27.5	32
Rottweiler	SD	9	27	1	4	222.2	46
	non-SD	20	27	0	19	40.9	19
Shetland Sheepdog	SD	15	24	0	3	339.2	75
	non-SD	19	25	0	20	33.6	16
Siberian Husky	SD	22	14	2	10	176.1	79
	non-SD	15	21	0	15	52.2	12
Standard Poodle	SD	13	26	1	7	215.6	87
	non-SD	17	44	0	36	25.3	40
Wolf	SD	34	25	3	16	207.7	95
	non-SD	25	28	0	14	36.3	32

Of the 403 distinct CNV regions, 143 are present in multiple individuals and 260 were identified in a single individual. Interestingly, approximately 80% of these "singletons" are located outside of SDs (Table 2) as has been observed in humans [20-22]. Moreover, CNV complexity was markedly different between SD and non-SD CNVs. Specifically, we define CNV regions that exhibit both gains and losses in copy number within a single individual as complex. While only 14 complex regions were identified, they are all from segmental duplications. These observations are consistent with the dynamic nature of SDs [17,20-26], which are likely to harbor CNVs that are polymorphic within and between breeds.

### CNV genotyping using a custom aCGH chip

**Figure 2:**
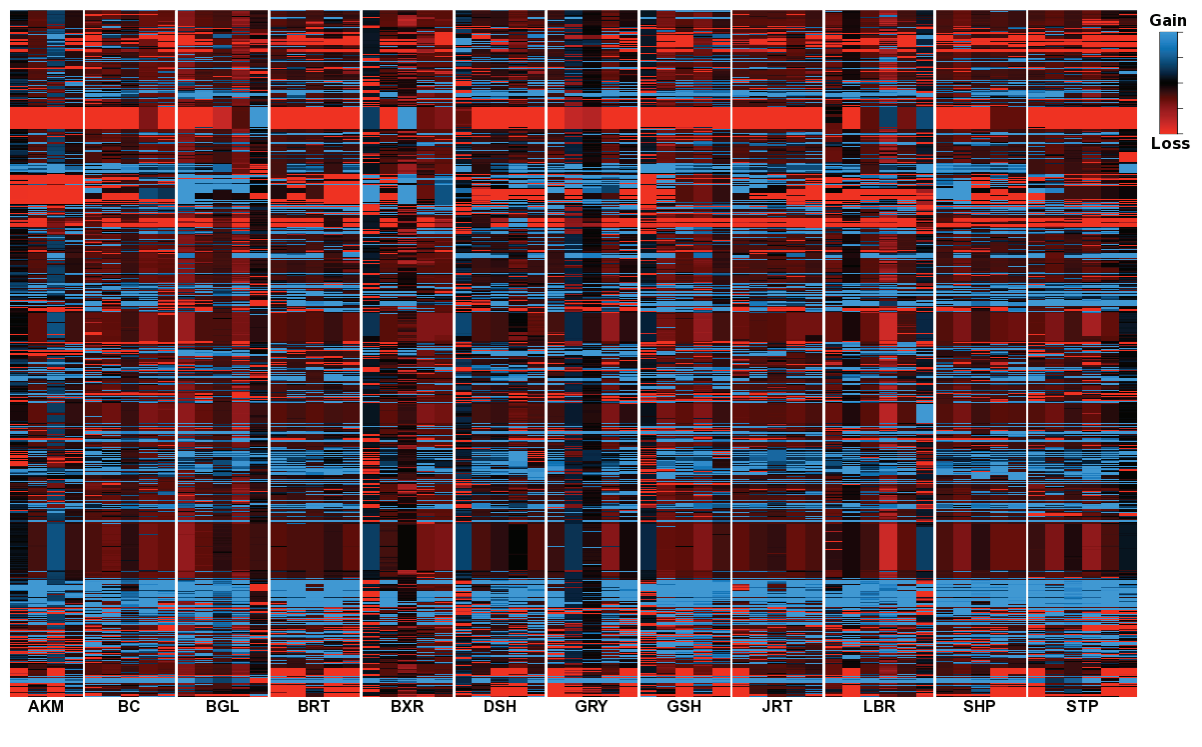
**Heatmap representation of CNVs in all individuals**. Columns represent individuals and rows represent a transformed measure of the posterior probability of each aCGH probe coming from a loss, normal copy, or gain state, denoted as P_Loss_, P_Normal_, and P_Gain_, respectively. Specifically, for each probe, the posterior probabilities of each state obtained from RJaCGH were converted into a single value by first dividing all three posterior probabilities by the largest value and then calculating a transformed score defined as (P_Gain _- P_Normal_) - (P_Loss _- P_Normal_), which results in a probe score that varies between -1 and 1. The values of -1, 0, and 1 correspond to the strongest evidence for loss, normal copy, and gains, respectively. Intermediate values reflect more uncertainty as to the state a given probe is in. Breeds are abbreviated as follows: Alaskan Malamute (AKM), Border Collie (BC), Beagle (BGL), Brittany (BRT), Boxer (BXR), Dachshund (DSH), Greyhound (GRY), German Shepherd (GSH), Jack Russell Terrier (JRT), Labrador Retriever (LBR), Shar Pei (SHP) and Standard Poodle (STP).

To better understand how CNV variation is apportioned within and between breeds, we designed a custom 12-plex NimbleGen aCGH chip and genotyped 61 dogs from 12 diverse breeds (Table 3) for all known canine CNVs (Figure 1). The average probe density was approximately 560 bp, and all of the hybridizations were performed with the same female Boxer used in previous aCGH experiments. We used a hidden Markov model implemented in the software package RJaCGH [27] to call CNVs for each CNV region in each sample (see Methods). The RJaCGH software package assigns a posterior probability to each aCGH probe as being in a gain, loss, or normal copy state. A summary of the posterior probabilities of each probe across all 61 individuals is shown in Figure 2.

**Table 3:** Summary of CNVs identified in each breed with the genotyping aCGH chip

Breed	N^a^	Total CNVs	Average^b ^	Range^c^	Average H_e_	Fixed Gains	Fixed Losses	Unique CNVs	Genic CNVs
Alaskan Malamute	4	406	188	86-306	0.194	15	22	8	194
Beagle	5	467	175	40-282	0.201	9	0	4	185
Border Collie	5	388	228	92-306	0.244	16	11	10	223
Boxer	5	403	133	72-244	0.160	6	0	7	165
Brittany	5	337	229	84-296	0.227	25	5	2	195
Dachshund	5	340	150	86-223	0.171	6	4	4	168
German Shepherd	5	382	201	144-219	0.193	26	18	5	196
Greyhound	5	394	156	40-267	0.180	7	6	6	189
Jack Russell Terrier	5	379	180	111-267	0.185	27	6	2	189
Labrador Retriever	6	409	179	119-254	0.194	8	7	7	206
Shar Pei	5	353	189	93-262	0.191	22	4	3	170
Standard Poodle	6	470	237	112-332	0.242	35	1	18	230

^a ^Denotes the number of individuals studied.

^b ^Average number of CNVs per individual

^c ^Indicates the range in the number of CNVs identified per individual within each breed.

Raw CNV calls from RJaCGH were filtered based on the number of data points, average posterior probabilities for probes in the putative CNV, and average log_2 _values (see Methods). Of the 892 regions studied, 665 (75%) had at least one individual containing a CNV. Over 95% of the CNV regions that appeared as monomorphic were previously identified in a breed not studied in the CNV genotyping panel; thus, failure to confirm CNVs in these regions is likely due to both individual or breed specific CNVs and false positives in previous CNV discovery experiments. As shown in Table 3, the average number of CNVs across all individuals was 187, ranging from 40 (in a Beagle and Greyhound) to 332 (in a Standard Poodle).

Before pursuing detailed population genetics inferences, we performed three analyses to assess data quality and false discovery rates. First, we performed three self-self hybridizations of a Boxer, Greyhound, and Shar-Pei. Using the same criteria to identify CNVs as described above, we called 0, 1, and 6 CNVs in the Shar-Pei, Boxer, and Greyhound, respectively. Thus, the self-self hybridizations suggest a low false discovery rate (< 5%). Second, we included 42 control regions on the genotyping aCGH chip selected from putatively single copy sequence defined from earlier CNV experiments [17]. Across all individuals, and thus a total 61 × 42 = 2,562 total control regions, only 56 CNVs were called (located in 14 distinct control regions), which also suggests a low false discovery rate. Note, it is plausible that genuine CNVs exist in some of these putative single copy control sequences, which were not observed in previous studies that examined a smaller number of individuals. Indeed, Monte Carlo simulations demonstrate that the expected number of control regions to harbor a CNV given 56 false positives is 31 (standard deviation = 2), suggesting that the observed patterns of CNVs in control regions are more clustered than expected by chance and hence some may be genuine CNVs. Third, three of the individuals included in the genotyping panel (a German Shepherd, Labrador Retriever, and Standard Poodle) were also previously interrogated for CNVs with the SD [17] and 2.1 chips (described above). The average overlap between CNVs called in the previous aCGH experiments and the genotyping chip across all three samples was 74.9%. To interpret the observed amount of overlap, we performed extensive simulations that recapitulate characteristics of the three aCGH chips and distribution of log_2 _values (see Methods). The observed overlap was similar to the simulated data (average overlap 71.9%, with a 95% confidence interval of 70.9-73.2%), and the discordances are primarily a result of different probe densities across chips that influences the power to detect CNVs. Overall, these three analyses suggest the CNV genotype data is of high quality.

Furthermore, we also examined whether CNV calls were more concordant between the genotyping chip and the SD chip or between the genotyping chip and NimbleGen 2.1 tiling array. In general, the concordances were similar, but higher for CNVs initially discovered on the SD chip (0.78) than CNVs discovered on the NimbleGen 2.1 tiling chip (0.71). Moreover, as expected, larger CNVs (> = 100 kb) were more concordant (81.6%) than smaller (< 100 kb) CNVs (74.9%).

### Patterns of CNV diversity within breeds

**Figure 3:**
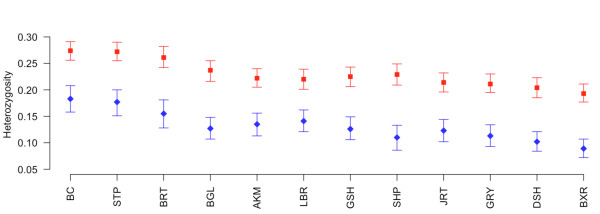
**Average heterozygosity of SD and non-SD CNVs**. Red squares and blue diamonds denote average heterozygosity for CNV regions associated with SDs and non-SDs, respectively. Vertical lines represent 95% confidence intervals. Breed abbreviations are described in Figure 2.

We estimated approximate allele frequencies for each breed and for each CNV using a simple EM algorithm [28] (see Methods). From these allele frequencies, we calculated the expected heterozygosity (H_e_) for each breed at every polymorphic CNV region, and the average H_e _for each breed is shown in Table 3. As expected from SNP and sequence data [1,3], Boxers were the least diverse breed studied and Border Collies were the most diverse breed (Table 3). Interestingly, we observe a significant difference (p < 10^-5^) in the average H_e _between CNVs from SDs and CNVs not from SD (Figure 3) in all breeds, consistent with the dynamic nature of SDs leading to increased segregating variation.

**Figure 4:**
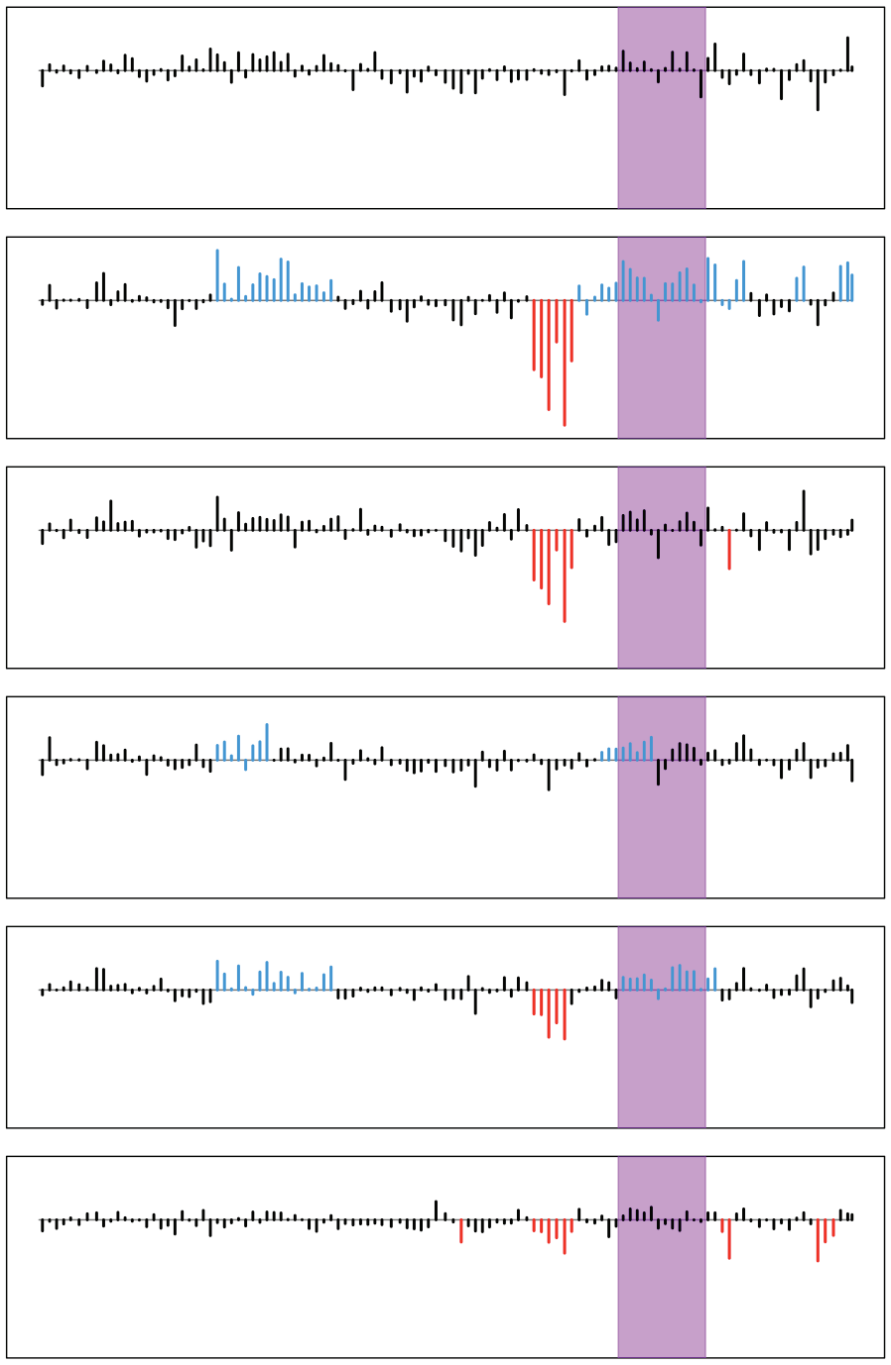
**Patterns of CNVs in six Standard Poodles for a region on CFA12**. Each bar represents the log_2 _value (y-axis) of a probe as a function of position (x-axis) across the region. Blue, red, and black bars indicate whether the probe was called as being in a gain, loss, or normal copy state, respectively. Highlighted in purple is a genic region corresponding to the location of the human homologs of the *PSORS1C2, CDSN*, and *CCHCR1 *genes. Note, the heterozygosity of this region in the main text is based on the entire region, and not just the purple highlighted interval.

To better understand how CNVs contribute to within breed diversity, we searched for CNV regions that exhibited high levels of heterozygosity. Interestingly, 45 regions were identified that exhibited high diversity in one or more breeds (H_e _> 0.6). For example, a CNV region on CFA12 was identified in the Standard Poodle, which contains a number of genes, such as *PSORS1C2, CDSN*, and *CCHCR1*, that are associated with various epithelial processes and skin disorders (Figure 4). Standard Poodles are a breed marked with common occurrences of skin disorders or disorders with epithelial symptoms such as Cushing's disease (hyperadrenocorticism) [29,30] and Sebaceous adenitis [31,32]. Additionally some skin disorders, such as psoriasis in humans, have been associated with copy number polymorphisms [33]. Thus, *PSORS1C2, CDSN*, and CCHCR1 are excellent candidates to pursue in future association studies of skin phenotypes in Standard Poodles. Furthermore, a topoisomerase gene, *TOP3B*, involved in the cutting of DNA strands during transcription and recombination [34], was also found to be polymorphic in six breeds (Alaskan Malamute, Border Collie, Brittany, Labrador Retriever, Shar Pei, and Standard Poodle).

### Patterns of CNV diversity between breeds

**Figure 5:**
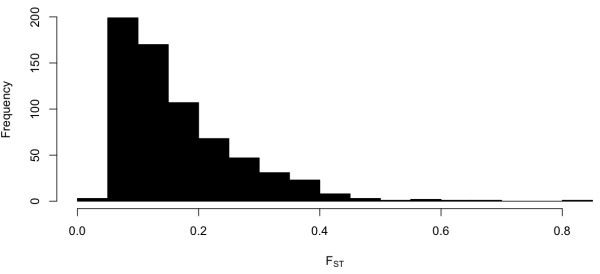
**Distribution of F_ST _from all polymorphic CNV regions**.

To better understand patterns of CNV variation between breeds, we calculated F_ST _for each polymorphic CNV region. The distribution of F_ST _across all CNV regions is shown in Figure 5, which ranges from 0.028 to 0.86. The average F_ST _is 0.168, which is comparable, although slightly lower than estimates of F_ST _in SNP data [4,8]. No significant difference in F_ST _was detected between SD and non-SD CNVs (p > 0.05). A number of interesting genes exist among the top 50 most differentiated CNV regions that may be relevant to phenotypic variation between breeds, such as *ATBF1*, a zinc finger transcription factor that regulates neuronal and muscle development [35] and *NKAIN2*, which is associated with susceptibility to lymphoma [36], the most common form of canine cancer [37].

**Figure 6:**
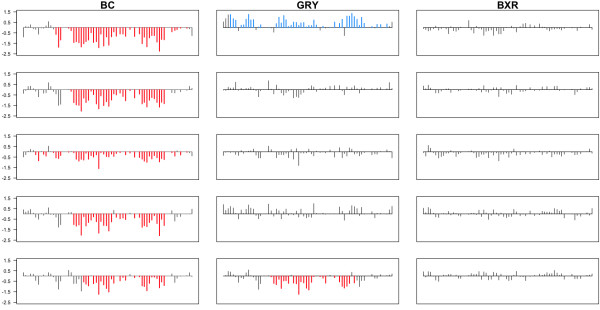
**Diverse CNV region on CFA 1**. Each bar represents the log_2 _value (y-axis) of a probe as a function of position (x-axis) across the region. Blue, red, and black bars indicate whether the probe was called as being in a gain, loss, or normal copy state, respectively. All Border Collie (BC) individuals have a loss in this region, all Boxer (BXR) individuals show no evidence for a CNV, and Greyhounds (GRY) segregate both gains and losses.

In addition, we also identified CNVs where all individuals within one or more breeds carried a duplication or deletion, but was absent in at least one of the remaining breeds. In total, 49 such regions exhibiting this pattern were identified (Figure 6, Additional file 2), 21 of which overlap the top 50 most differentiated CNVs described above. A number of these divergent regions possessed genes that potentially contribute to phenotypic differences between breeds such as development (*OBSCN, NOTCH2*, and *NKD2*), neuronal processes (*TNFRSF1B *and *ATBF1*), olfaction (*OR4S2, OR4C30*, and *OR52B4*), and metabolism (*HMGCS2*).

## Conclusions

In summary, we have compiled the most comprehensive catalog of canine structural variation described to date. Moreover, we examined patterns of variation for all known canine CNVs in a diverse panel of 12 breeds, providing the first insight into how structural variation is apportioned within and between breeds. Interestingly, we found high levels of CNV diversity within breeds, suggesting that structural variation may be an important source of genetic variation contributing to within breed patterns of phenotypic diversity. Moreover, our data is consistent with a high rate of de novo CNV formation within breeds. We anticipate that the CNV resources developed in this work will complement existing genome-wide panels of SNP markers [1,3,9] by providing the foundation for future association studies to delimit how structural variation contributes to canine phenotypic variation and disease susceptibility.

## Methods

### DNA samples

For the genome-wide tiling aCGH experiments, a single individual from the following breeds was used: Basenji, Doberman, German Shepherd, Labrador Retriever, Pug, Shetland Sheepdog, Siberian Husky, Standard Poodle, Rottweiler, and a Grey Wolf. Samples used in the genotyping aCGH experiments included the following breeds: Alaskan Malamute, Beagle, Border Collie, Boxer, Brittany, Dachshund, German Shepherd, Greyhound, Jack Russell Terrier, Labrador Retriever, Shar Pei, and Standard Poodle. A total of 3 "self-self" hyrbidizations were performed using the female Boxer reference, a Greyhound, and Shar Pei. DNA quality of all samples was assessed by taking OD260/280 and OD260/230 readings using a nanospectrometer.

### aCGH and CNV identification

The high density aCGH chip was designed and produced by NimbleGen http://www.NimbleGen.com, and included 2,164,508 oligonucleotide probes with an average probe spacing of 1050 bp. All genomic DNA samples were sent to NimbleGen who performed the hybridizations. In all cases a female Boxer was used as the reference sample. Each hybridization was initially subjected to segmentation using the CGH-segMNT program within the NimbleScan software package. Segments were further partitioned if there was a gap greater than 50 kb between adjacent probes. Furthermore, segments within 5 kb of one another and with consistent log_2 _ratios (either both positive or both negative) were merged together to form a new segment. To define segments corresponding to gains and losses, we developed an adaptive threshold algorithm that takes advantage of the observation that segments with more data points require smaller changes in log_2 _ratios to be reliably called as a gain or loss whereas segments with fewer data points require larger magnitudes of log_2 _ratios to be accurately called as a gain or loss. We trained our algorithm on the self-self hybridization to identify parameters resulting in a low false discovery rate. Specifically, if a segment contained 5-10 data points, 11-100, or > 100 data points, we required an average log_2 _ratio that was 3, 2, and 1 standard deviations or greater from the mean, respectively, to be retained. Thus, a minimum of five probes was required to call a CNV. All aCGH data has been submitted to GEO http://www.ncbi.nlm.nih.gov/geo/ under accession number GSE26170.

### CNV genotyping

A custom aCGH genotyping chip was developed with NimbleGen using the CamFam2.0 assembly. The chip contains 12 individual lanes, each spotted with 136,929 oligonucleotide probes with a mean probe spacing of approximately 560 bp. These probes were primarily designed to tile over all previously identified CNVs including the 678 CNV regions identified in segmental duplications [17], 403 CNV regions identified from a genome-wide CNV detection survey using the NimbleGen 2.1 tiling arrays, and 60 CNV regions from a separate genome-wide study [10]. In addition, 42 putative single copy control regions that had never before been found to contain CNVs and were not associated with segmental duplications were included. Finally, 1,095 additional regions were included on the chip, which were derived from lower confidence CNV calls. Note, these CNV regions were excluded in all analyses described in this manuscript, but information about them is provided in Additional files 3 and 4. Coordinates from all these regions were merged and covered with aCGH probes. Hybridizations of 61 individuals from 12 different breeds were performed using a common female Boxer as a reference sample. Additionally, three self-self hybridizations were also performed. Breeds were randomized across chips to mitigate confounding factors.

The raw log_2 _ratios were first normalized by loess regression. Next, we fit linear models to the residuals of the loess regression to account for spot position and chip number. For all samples, individual probes were grouped into sets of five continuous probes (unless adjacent probes were more than 5 kb apart) and their log_2 _value was averaged. The average log_2 _values were then called for CNVs using a reversible jump hidden Markov Model implemented in the software RJaCGH [27]. The output of RJaCGH consists of a state call for each probe (i.e., gain, normal copy, and loss) and the posterior probability of being in each state. Using the self-self hybridizations, adaptive thresholds were established to filter these raw CNV calls based on the number of data points, average posterior probabilities for probes in the putative CNV, and average log_2 _value across probes in a putative CNV. Specifically, for segments consisting of three to five averaged data points (corresponding to approximately 8.4 - 14 kb), we required a posterior probability greater than 0.75 and a log_2 _value equal to the mean ± 0.5*standard deviation of all log_2 _values (note, plus for gains and minus for losses). If the segment consisted of > 5 averaged data points (corresponding to a minimum size of approximately 16.8 kb), we retained RJaCGH CNV calls with a posterior probability ≥ 0.6. All unique X-linked CNVs called as deletions in male dogs were removed since the reference was a female dog.

### Simulations

Simulations were performed to interpret the observed amount of overlap between CNVs for the German Shepherd, Labrador Retriever, and Standard Poodle samples, which were analyzed on multiple chip platforms. The aCGH designs considered included the custom segmental duplication chip [17], the genome-wide 2.1 million feature chip, and the genotyping chip. Distributions of CNV sizes, probe spacing, and log_2 _values were generated for gains, normal copy, and losses conditional on the observed distributions of these quantities in each sample. Using this information, normal copy and CNV regions were simulated for each sample across all three array platforms, and subjected to the same CNV analysis as described above. For a given region, overlapping CNV calls are defined in cases where the same CNV genotype is obtained between platforms.

### CNV allele frequency estimations

Exact allele frequencies are difficult to calculate because precise copy numbers are unknown. To this end, we inferred approximate allele frequencies by simplifying CNV phenotypes into three categories: normal copy, gain, or loss. The frequency of each category was estimated by a standard EM algorithm [28]. The estimated allele frequencies were used to calculate expected heterozygosity (H_e_) for each breed and each CNV region as H_e _= 1 - (p^2 ^+ q^2 ^+ r^2^), where p, q, and r denote the frequencies of chromosome carrying normal copy, gains, and losses, respectively. Similarly for each CNV region, we calculated F_ST _as: F_ST _= 1 - h_s _/h_t_, where h_s _and h_t _denote average heterozygosity within subpopulations (breeds) and total heterozygosity, respectively.

### Gene identification and PANTHER analysis

A catalog of all canine peptides was downloaded from Ensembl ftp://ftp.ensembl.org/pub/current_fasta/canis_familiaris/pep/, which contains 25,546 peptides. For each breed, the total number of genic CNVs and associated peptides were determined and PANTHER Molecular Function terms were assigned to all peptides using the PANTHER Hidden Markov Model scoring tools http://www.pantherdb.org/downloads/. PANTHER Molecular Function terms with less than five observations among the breed associated genes were not analyzed further. For each breed, we tested for overrepresentation of PANTHER terms in the CNV regions using the hypergeometric distribution. Bonferroni corrections were used to correct p-values for multiple hypothesis testing.

## Competing interests

The authors declare that they have no competing interests.

## Authors' contributions

TJN, EEE, and JMA conceived of and designed the experiments. TJN and CB performed all of the experiments. TJN and JMA analyzed the data. TJN and JMA wrote the paper. All authors read and approved the final manuscript.

## Supplementary Material

Additional file 1 **Enriched Panther Molecular Function Terms in CNV regions identified on the 2.1 chip**. This table summarizes Gene Ontology Molecular Function terms that are significantly overrepresented in CNV regions identified on the 2.1 chip. Click here for file

Additional file 2 **Heterozygosities of the 49 regions where one breed was fixed for a CNV that was absent in one or more breeds**. This table summarizes heterozygosities of the 49 CNV regions that exhibit interesting patterns of allele frequency variation within and between breeds. Click here for file

Additional file 3 **Summary of all CNV regions**. This table provides information on the genomic locations and sources for all CNV regions. Click here for file

Additional file 4 **CNV genotypes**. This table summarizes genotypes for all individuals across all CNVs. Click here for file
