# Supplementary material for: A high-resolution integrated map of copy number polymorphisms within and between breeds of the modern domesticated dog
Source: BMC Genomics. 2011 Aug 16;12:414. doi: 10.1186/1471-2164-12-414 (PMC3166287; doi:10.1186/1471-2164-12-414)
Supplement: Additional file 1 — Enriched Panther Molecular Function Terms in CNV regions identified on the 2.1 chip. This table summarizes Gene Ontology Molecular Function terms that are significantly overrepresented in CNV regions identified on the 2.1 chip. [file 1471-2164-12-414-S1.PDF]

Supplementary Table 1. Enriched Panther Molecular Function Terms in CNV regions identified on the 2.1 chip.

Term		
**All**	**N[a]**	**P-Value[b]**
Defense/immunity protein	50	1×10^{-6}
Molecular function unclassified	422	1×10^{-6}
Oxidoreductase	13	9.1×10^{-3}
Protease	6	1.7×10^{-2}
Receptor	11	4.2×10^{-2}
Signaling molecule	19	2.1×10^{-3}
Transporter	11	1.8×10^{-2}
**Located in SDs**		
Defense/immunity protein	49	1×10^{-6}
Molecular function unclassified	262	1.2×10^{-6}
Oxidoreductase	13	4.2×10^{-4}
Signaling molecule	14	1.5×10^{-3}
**Not located in SDs**		
Molecular function unclassified	160	1×10^{-6}
Receptor	8	3.2×10^{-3}
Select regulatory molecule	9	1.5×10^{-2}
Signaling molecule	5	3.6×10^{-2}
Transferase	7	2.1×10^{-2}
Transporter	6	6.1×10^{-3}

[a] Number of genes annotated with a given Panther term.

[b] Bonferroni corrected P-values.
