# Supplementary material for: A high-resolution integrated map of copy number polymorphisms within and between breeds of the modern domesticated dog
Source: BMC Genomics. 2011 Aug 16;12:414. doi: 10.1186/1471-2164-12-414 (PMC3166287; doi:10.1186/1471-2164-12-414)
Supplement: Additional file 2 — Heterozygosities of the 49 regions where one breed was fixed for a CNV that was absent in one or more breeds. This table summarizes heterozygosities of the 49 CNV regions that exhibit interesting patterns of allele frequency variation within and between breeds. [file 1471-2164-12-414-S2.PDF]

Supplementary Table 2. Heterozygosities of the 49 regions where one breed was fixed for a CNV that was absent in one or more breeds.

CNV Region	AKM	BCC	BGL	BRT	BXR	DSH	GRY	GSH	JRT	LBR	SHP	STP	FST
chr1:105809893-105848142	0.232	0.165	0.19	0.19	0	0.35	0.358	0.466	0.466	0.165	0.35	0.486	0.398
chr3:34147139-34446301	0.232	0	0.358	0.545	0.19	0.19	0.35	0.358	0.19	0.486	0.497	0.62	0.232
chr4:61441171-61463512	0.433	0.304	0.466	0.165	0.35	0.35	0.19	0.35	0.35	0.3	0	0.415	0.265
chr4:77649573-77679587	0.5	0.165	0.35	0	0	0.35	0.466	0.165	0.466	0.488	0.35	0.488	0.336
chr5:81143006-81475905	0	0.466	0.165	0.19	0.19	0.165	0.495	0.165	0.165	0.165	0.466	0.3	0.507
chr7:46759465-46785299	0.232	0.358	0.497	0.35	0.19	0.165	0.19	0	0.19	0	0.19	0.529	0.352
chr7:70556322-70584206	0.415	0.466	0.497	0.19	0.35	0.466	0.19	0.165	0.466	0.415	0	0.3	0.238
chr8:59108084-59139186	0.415	0.466	0.35	0	0	0.19	0.19	0.165	0.19	0.3	0	0	0.411
chr8:76331651-77310517	0.165	0.165	0.497	0.466	0	0.495	0.165	0.509	0.165	0.486	0.165	0.3	0.379
chr11:12484835-12611952	0.232	0.35	0.35	0.35	0.35	0.19	0.466	0.495	0.165	0.486	0	0.488	0.27
chr11:43753544-43803409	0.584	0	0.466	0.584	0.466	0.654	0.35	0.466	0.358	0.488	0.35	0.578	0.149
chr15:32826968-32851381	0.165	0.165	0.466	0	0	0.19	0.495	0	0.19	0	0.466	0.159	0.558
chr16:44333540-44370693	0.232	0.35	0.495	0.35	0.35	0.35	0	0.19	0.35	0.3	0.165	0.159	0.295
chr17:64662082-64711046	0.5	0.466	0.495	0.495	0.35	0.495	0.165	0.165	0.165	0.486	0.19	0	0.338
chr18:5569059-5592288	0.232	0.466	0.19	0.165	0.35	0.19	0.35	0	0.495	0.159	0.466	0.488	0.298
chr18:7724276-7788068	0	0.35	0.19	0	0	0	0	0.165	0	0.159	0	0.3	0.566
chr19:22400293-22510098	0.415	0.466	0.466	0.495	0	0	0.19	0.165	0.19	0	0.466	0.159	0.373
chr21:29706307-29779822	0.415	0.466	0.358	0.466	0	0.358	0.35	0.165	0.495	0.509	0	0.529	0.319
chr21:30160320-30180764	0.415	0.165	0.35	0	0	0.19	0.19	0.466	0.497	0.159	0.466	0.3	0.338
chr21:31548110-31613003	0.5	0	0.466	0.495	0	0.35	0.466	0.165	0.35	0.415	0	0.3	0.31
chr23:23532155-23793196	0.232	0.495	0.165	0.165	0	0.495	0.165	0.165	0.165	0.486	0.495	0.165	0.401
chr25:48051062-48118154	0.5	0.19	0.35	0.19	0	0.35	0.358	0.35	0.165	0.542	0	0.578	0.307
chr26:15764819-15784418	0	0.495	0.165	0.466	0	0.165	0.19	0	0.165	0.159	0	0	0.668
chr33:5280295-5301393	0.415	0.35	0.35	0	0.19	0.35	0.466	0.165	0.19	0.415	0	0.415	0.285
chr36:7018416-7077514	0.232	0	0	0.165	0	0	0	0	0	0.3	0	0.159	0.628
chrUN:8774774-8804891	0.415	0.466	0.584	0.495	0	0	0	0.165	0.19	0.159	0.495	0.3	0.362
chrUN:8860924-8909099	0.415	0.466	0.497	0.495	0	0	0.19	0.165	0.19	0	0.35	0.3	0.356
chrUN:8954918-	0.5	0.495	0.584	0.495	0	0.19	0.35	0.165	0.466	0.159	0.495	0.415	0.263

9099047													
chrUN:9104251-9128197	0.415	0.466	0.497	0.495	0	0	0.35	0.495	0.19	0.159	0.495	0.488	0.194
chrUN:9322911-9572289	0.5	0.495	0.545	0.495	0	0.495	0.35	0.165	0.584	0.415	0.165	0.486	0.241
chrUN:9583039-9605370	0.5	0.466	0.584	0.495	0	0	0.35	0.165	0.35	0.159	0.495	0.415	0.291
chrUN:29067064-29095726	0	0.584	0.35	0.19	0.35	0.358	0.19	0.35	0	0.488	0.466	0.486	0.185
chrUN:39827115-39865925	0.232	0	0.19	0.35	0.35	0.19	0.466	0.165	0.466	0.486	0.466	0.3	0.284
chrUN:42463731-42484892	0.232	0.35	0.35	0	0.35	0.35	0.35	0.165	0.19	0.488	0	0.488	0.292
chrUN:44129785-44184590	0.433	0.165	0.466	0.495	0.19	0.35	0.35	0.495	0	0.486	0.19	0.578	0.261
chrUN:47328709-47353237	0.5	0.466	0.584	0.495	0	0	0.19	0.165	0.35	0.159	0.495	0.415	0.311
chrUN:47767042-47804896	0.232	0.578	0.466	0.495	0.35	0.466	0.35	0.497	0	0.431	0.35	0.488	0.103
chrUN:48300375-48320943	0.232	0.35	0.466	0.35	0	0.19	0.19	0	0.19	0	0.35	0.415	0.107
chrUN:49688572-49728548	0.415	0.358	0.466	0.495	0	0.35	0.165	0.495	0.19	0.3	0.466	0.486	0.252
chrUN:49825717-49846170	0.415	0.495	0.584	0.495	0	0	0.19	0.165	0.35	0	0.495	0.415	0.342
chrUN:50574679-50613550	0.579	0.19	0.19	0.466	0	0.35	0	0.165	0.35	0.159	0.495	0.3	0.381
chrUN:56026391-56046557	0	0.35	0	0.19	0.35	0	0	0.19	0	0.3	0.35	0.486	0.237
chrUN:56996651-57021031	0	0.497	0.35	0.358	0.35	0.35	0.495	0.19	0.19	0.488	0.35	0.165	0.336
chrUN:58180216-58200800	0.415	0.495	0.584	0.495	0	0.19	0.35	0.165	0.19	0	0.466	0	0.368
chrUN:59841790-59870497	0.165	0.466	0.19	0.19	0.165	0.35	0.19	0.466	0.35	0.3	0.165	0	0.483
chrUN:70894231-70914413	0	0.495	0.35	0.165	0.19	0.466	0.466	0.165	0.466	0.486	0.19	0.415	0.342
chrUN:73369676-73388095	0.5	0.35	0.495	0.19	0.466	0.495	0.165	0.495	0.35	0.159	0.35	0	0.282
chrX:43126076-43151226	0	0.165	0.584	0.19	0.19	0.35	0.19	0.466	0.495	0.3	0	0	0.369

Breed abbreviations are as follows: Alaskan Malamute (AKM), Border Collie (BCC), Beagle (BGL), Brittany (BRT), Boxer (BXR), Dachshund (DSH), Greyhound (GRY), German Shepherd (GSH), Jack Russell Terrier (JRT), Labrador Retriever (LBR), Shar Pei (SHP), and Standard Poodle (STP).
